# Salary Differences by Gender, Race, and Ethnicity Among Assistant Professors at US Medical Schools

**DOI:** 10.1001/jamanetworkopen.2025.9583

**Published:** 2025-05-14

**Authors:** Dalia Owda, Michael O. Mensah, David Yang, Maureen E. Canavan, Cary P. Gross, Sarwat I. Chaudhry

**Affiliations:** 1 Department of Emergency Medicine, Henry Ford Health System, Detroit, Michigan; 2 Department of Psychiatry, Yale School of Medicine, New Haven, Connecticut; 3 Department of Emergency Medicine, Yale School of Medicine, New Haven, Connecticut; 4 Cancer Outcomes Public Policy and Effectiveness Research (COPPER) Center, Yale School of Medicine, New Haven, Connecticut; 5 Section of General Internal Medicine, Department of Medicine, Cancer Outcomes Public Policy and Effectiveness Research (COPPER) Center, Yale School of Medicine, New Haven, Connecticut; 6 Center for Outcomes Research and Evaluation, Yale-New Haven Health System, New Haven, Connecticut; 7 Section of General Internal Medicine, Department of Medicine, Yale School of Medicine, New Haven, Connecticut

## Abstract

**Importance:**

Salary inequities by gender are well documented in medicine, but there is limited understanding of racial and ethnic disparities or how these intersect with gender across clinical specialties.

**Objective:**

To determine whether salaries for assistant professors at US medical schools differ by gender, race and ethnicity, and gender-race-ethnicity intersections across clinical specialties.

**Design, Setting, and Participants:**

This cross-sectional study of medical school assistant professor faculty salary data assessed 19 clinical specialties by gender, race and ethnicity, and gender-race-ethnicity intersections. Aggregated data were obtained from the Association of American Medical Colleges Faculty Salary Report for 2022 to 2023 for assistant professors at 153 US medical schools.

**Exposures:**

Gender, race and ethnicity, and gender-race-ethnicity.

**Main Outcomes and Measures:**

Median annual salary and salary ratios were calculated for assistant professors across gender, race and ethnicity, and at their intersections for the 2022-2023 academic year. Salary ratios compared women with men, Asian and underrepresented in medicine (URIM) faculty to White faculty, and various gender-race-ethnicity subgroups to White men.

**Results:**

In this cross-sectional study of 45 906 assistant professor faculty members from 19 clinical specialties in 2022 and 2023, there were 23 538 (51%) men and 22 368 (49%) women, with a racial and ethnic composition of 10 294 (27%) Asian, 4543 (12%) URIM, and 23 781 (62%) White. Across all specialties, women (median annual salary, $266 450; salary ratio, $0.81:$1.00) earned less than men (annual median salary, $330 000). Asian (median annual salary, $291 360; salary ratio, $0.97:$1.00) and URIM (median annual salary, $278 010; salary ratio, $0.93:$1.00) physicians were paid less than White physicians (median annual salary, $300 000). Intersectional analyses showed that URIM women were paid the least (median annual salary, $259 570; salary ratio, $0.78:$1.00) compared with White men (median annual salary, $333 800).

**Conclusions and Relevance:**

This cross-sectional study of assistant professor faculty across 19 clinical specialties found salary disparities, with Asian and URIM, both men and women, receiving lower pay than White men. These findings suggest the need to address salary inequities in academia using an intersectional approach that considers both gender and race and ethnicity.

## Key Points

**Question:**

Are there salary differences for assistant professors at US medical schools as assessed by gender, race and ethnicity, and gender-race-ethnicity intersections?

**Findings:**

In this cross-sectional study of 45 906 assistant professor faculty members across 19 clinical specialties, women physicians had lower salaries than men, and Asian and underrepresented in medicine (URIM) physicians had lower salaries than White physicians. Observed salary differences increased at gender-race-ethnicity intersections, with URIM women having the lowest salaries compared with White men.

**Meaning:**

These findings suggest that salary inequities by gender, race and ethnicity, and gender-race-ethnicity intersections persist across specialties for assistant professors at US medical schools, reflecting broader systemic issues in academic medicine.

## Teaser

This cross-sectional study evaluates whether salary differences exist by gender, race and ethnicity, or gender-race-ethnicity intersections among assistant professors at US medical schools.

## Introduction

In 2022, women represented 47% of the US active workforce.^1^ Black and Hispanic workers also made up a substantial and increasing portion of the active workforce, including one-quarter of the active science, technology, engineering, and mathematics workforce.^2,3,4^ With this increasing representation of women and racial and ethnic groups in the workforce, gender pay disparities exist, with women earning $0.84 to every dollar earned by men in the overall workforce.^1^ The pay gap is even wider for women of racial and ethnic minoritized groups compared with White men, with Black women earning $0.63 per dollar, Latina women $0.58, and Asian women $0.78, although variations exist among Asian subgroups.^4^

Prior studies have revealed gender salary disparities among physician-scientists, department chairs, and medical school faculty^5,6,7^ and in several specialties, such as surgery, internal medicine, emergency medicine, and ophthalmology.^8,9,10,11^ Salary disparities by race and ethnicity have been observed in pediatrics.^12^ There are limited data, however, on salary disparities at gender-race-ethnicity intersections across clinical specialties. Without these data, we are likely to miss important inequities in faculty salaries that may disproportionately affect certain subgroups.

Additionally, it is important to consider salary inequities within the larger context of diversity, equity, and inclusion efforts. Prior studies have shown that higher-paying specialties, such as surgical specialties, are less successful at recruiting women and URIM trainees.^13,14^ Beyond recruitment, no studies to date have examined whether the demographic diversity of faculty is associated with salary differences. Our aims were (1) to determine whether assistant professor faculty salaries across specialties differed by gender, race and ethnicity, and gender-race-ethnicity, and (2) to investigate whether there was an association between the faculty demographic composition of a clinical specialty and its salary differences.

## Methods

### Design and Data Source

We performed a cross-sectional analysis of faculty salaries at the assistant professor rank by gender, race and ethnicity, and gender-race-ethnicity for 19 clinical specialties. This study was deemed exempt from review by the Yale School of Medicine institutional review board because all data were deidentified. Additionally, informed consent was not required for this study as it used publicly available, deidentified data, ensuring that no personal or identifiable information about participants was accessed or used. This study was reported following the Strengthening the Reporting of Observational Studies in Epidemiology (STROBE) reporting guideline.

These specialties included 11 nonsurgical specialties (anesthesiology, dermatology, family medicine, internal medicine, clinical pathology, pediatrics, psychiatry, radiology, emergency medicine, neurology, and physical medicine and rehabilitation), and 8 surgical specialties (obstetrics and gynecology, general surgery, neurosurgery, orthopedic surgery, plastic surgery, urology, ophthalmology, and otolaryngology). We selected these 19 specialties as they had the highest representation and data available by gender, race and ethnicity, and/or gender-race-ethnicity in the 2022-2023 Association of American Medical Colleges (AAMC) Faculty Salary Report.^15^ The study focused on assistant professors, using median compensation to reflect typical salaries, as this rank is a common entry point into academia and sets the pace for future earnings.^16^

We obtained assistant professor faculty salaries by gender, race and ethnicity, and gender-race-ethnicity from the 2022-2023 AAMC Faculty Salary Report.^15^ The survey is sent to the principal business officer at Liaison Committee on Medical Education–accredited, US-based medical schools and is typically completed by administrative staff at each medical school. The participating institutions self-report gender, race, and ethnicity for each faculty member. The report publishes data annually and includes both mean and median compensations and the number of faculty respondents in each specialty by rank, gender, race and ethnicity, and gender-race-ethnicity. The AAMC defines salary as “the sum of fixed/contractual salary, bonus/incentive pay, medical practice supplement, and uncontrolled outside earnings.”^15^ The 2022-2023 survey reflected data from 153 medical schools and had a 99% response rate.^5,15^

In the AAMC survey, gender categories were man or woman. Race and ethnicity categories were American Indian or Alaska Native; Asian; Black or African American; Hispanic, Latino, or of Spanish origin; multiple race, Hispanic; multiple race, non-Hispanic; Native Hawaiian or Other Pacific Islander; White; and other. The other category represented faculty members for whom none of the provided racial and ethnicity categories applied. Gender-race-ethnicity included combinations of the gender and race and ethnicity categories. Compensation statistics were reported by the AAMC when there were 5 or more observations in a specialty for each gender, race and ethnicity, or gender-race-ethnicity combination.

We categorized data from the AAMC Faculty Salary Report by gender (ie, man and woman); race and ethnicity (our categories included Asian, underrepresented in medicine [URIM], and White); and gender-race-ethnicity (ie, combinations of gender and race and ethnicity categories). We classified racial and ethnic groups as URIM according to the AAMC definition, which includes individuals who identify as American Indian or Alaska Native; Black or African American; Hispanic, Latino or of Spanish Origin; Multiple race, Hispanic; and Native Hawaiian or Other Pacific Islander.^17^ We calculated median salaries for the URIM group by weighting salaries based on the number of respondents in each URIM subgroup. To ensure data reliability and generalizability, we analyzed only specialties with at least 30 survey respondents.

For the second aim of this study, to examine the association between faculty demographics and salary differences, we used the 2022 AAMC Faculty Roster.^18^ This dataset covers the same clinical specialties as the 2022-2023 AAMC Faculty Salary Report except for neurosurgery, plastic surgery, and urology. Thus, our analysis included the 11 aforementioned nonsurgical specialties and 5 surgical specialties (obstetrics and gynecology, general surgery, orthopedic surgery, ophthalmology, and otolaryngology) at the assistant professor level. We again categorized racial and ethnic groups in the URIM category based on the AAMC definition.^17^

### Outcomes

Our primary outcomes were median compensation (rounded to nearest $10), absolute salary difference, and salary ratio. We compared these across specialties by gender, by race and ethnicity, and at their intersection, using men, White, and White men as the reference categories, respectively. Absolute salary differences were reported to directly compare dollar amounts.^19^

For salary ratios by gender, we divided the median salary for women by the median salary for men. For race and ethnicity salary ratios, we divided the median salary for each racial and ethnic subgroup (Asian, URIM) by the median salary for White faculty. For salary ratios by gender-race-ethnicity, we divided the median salary for each gender-race-ethnicity subgroup (Asian men, Asian women, URIM men, URIM women, and White women) by the median salary for White men.

For the secondary aim, we calculated percentages of physicians by gender, race and ethnicity, and their intersections across the 16 aforementioned specialties (11 nonsurgical and 5 surgical). We divided the count of each group by the total faculty count in each specialty. For URIM categories, we combined counts of racial and ethnic groups that comprised URIM as defined by the AAMC and divided by the total faculty count in each specialty.

### Interpretation of Salary Differences

We interpreted observed salary differences as potential inequities due to our study design. We focused on assistant professors in specific clinical specialties, controlling for career stage and specialty-specific factors. In an equitable system, we would expect no significant salary differences among this group. We considered statistically significant differences by gender, race and ethnicity, and their intersections as potential indicators of systemic inequities. While acknowledging possible unmeasured factors, we believe the consistency of findings across specialties could reveal potential inequities in academic medicine compensation.

### Statistical Analysis

Statistical analysis was performed using Stata, version 16.0 (StataCorp LLC). We calculated descriptive statistics, including median compensation, absolute salary differences, and salary ratios across specialties, grouped by gender, race and ethnicity, and their intersections. The analysis used aggregated data from the AAMC Faculty Salary Report, which did not provide sufficient granularity to calculate uncertainty indicators such as confidence intervals or standard errors.

For the secondary aim, we used 2-sided Spearman rank correlations to assess associations between faculty demographics and salary differences. We correlated group representation with salary ratios for each of the following gender, race and ethnicity, and gender-race-ethnicity categories: women, Asian, URIM, Asian men, Asian women, URIM men, URIM women, and White women. We excluded data with fewer than 30 respondents.

A correlation coefficient with an absolute value between 0 and 0.3 indicated a weak correlation; 0.31 and 0.7, a moderate correlation; and higher than 0.7, a strong correlation.^20^ For all statistical tests, a 2-sided *P* ≤ .05 was considered statistically significant.

## Results

### Sample Characteristics

This cross-sectional study included 45 906 assistant professor faculty members across 19 clinical specialties at 153 US medical schools in 2022 and 2023. The sample comprised 23 538 men (51%) and 22 368 women (49%) and included 10 294 Asian (27%), 4543 URIM (12%), and 23 781 White (62%) faculty members.

We analyzed data from a total of 45 906 faculty members by gender, 38 618 by race and ethnicity, and 38 527 by gender-race-ethnicity. There were 7288 faculty members for which race and ethnicity data were not available, and 7379 for which gender-race-ethnicity data were not available (Table 1 and Table 2).

**Table 1.** Median Salary and Salary Ratios for Assistant Professor Faculty Across Specialties by Gender and Race and Ethnicity

Specialty	Gender					Race and ethnicity							
	Men		Women			Asian			URIM			White	
	Median, $	No. (%)	Median, $	No. (%)	Ratio	Median, $	No. (%)	Ratio	Median, $	No. (%)	Ratio	Median, $	No. (%)
Overall^a^	330 000	23 538 (51)	266 450	22 368 (49)	0.81	291 360	10 294 (27)	0.97	278 010	4543 (12)	0.93	300 000	23 781 (62)
Total 19 clinical specialties^a^^,^^b^	340 800	21 681 (51)	278 020	20 980 (49)	0.82	304 940	9672 (27)	0.97	289 220	4177 (12)	0.92	314 180	22 262 (62)
**Nonsurgical specialties**													
Anesthesiology	457 050	1969 (62)	427 280	1193 (38)	0.93	446 690	712 (26)	1.00	443 750	293 (11)	0.99	448 920	1745 (63)
Dermatology	389 120	181 (37)	353 460	308 (63)	0.91	367 160	83 (20)	0.97	337 680	36 (9)	0.89	379 100	303 (72)
Family medicine	250 870	979 (46)	232 150	1164 (54)	0.93	243 970	313 (17)	1.01	233 240	350 (19)	0.96	242 670	1214 (65)
Internal medicine	292 450	7547 (54)	253 230	6468 (46)	0.87	274 500	4035 (35)	1.01	266 720	1315 (11)	0.98	271 770	6454 (55)
Clinical pathology	242 810	202 (53)	246 880	182 (47)	1.02	242 000	91 (30)	0.99	246 510	32 (10)	1.01	243 790	184 (60)
Pediatrics	238 160	2096 (31)	216 400	4561 (69)	0.91	227 000	1329 (24)	1.01	213 130	704 (12)	0.95	223 770	3605 (64)
Psychiatry	255 710	1021 (47)	249 290	1165 (53)	0.97	256 000	447 (24)	1.02	251 220	266 (14)	1.00	251 060	1144 (62)
Radiology	450 140	1769 (67)	425 720	887 (33)	0.95	433 910	695 (31)	0.96	445 530	168 (8)	0.99	451 000	1344 (61)
Emergency medicine	327 820	1135 (55)	311 120	930 (45)	0.95	318 980	278 (15)	0.99	311 870	207 (11)	0.97	320 830	1396 (74)
Neurology	259 570	916 (53)	241 940	797 (47)	0.93	252 060	435 (31)	1.03	250 680	146 (10)	1.02	244 870	840 (59)
Physical medicine and rehabilitation	282 730	323 (55)	242 570	266 (45)	0.86	261 120	132 (26)	0.97	273 570	65 (13)	1.02	268 420	302 (61)
**Surgical specialties**													
Obstetrics and gynecology	349 700	472 (23)	320 090	1582 (77)	0.92	313 050	259 (15)	0.94	308 540	289 (17)	0.93	332 090	1175 (68)
General surgery	421 200	455 (61)	368 510	291 (39)	0.87	377 700	140 (23)	0.94	372 650	53 (9)	0.93	401 740	407 (68)
Neurosurgery	679 450	453 (85)	600 000	79 (15)	0.88	659 620	115 (26)	0.98	671 690	36 (8)	1.00	674 050	286 (65)
Orthopedic surgery	559 990	913 (81)	455 610	221 (19)	0.81	495 980	152 (16)	0.90	477 300	72 (8)	0.87	550 710	720 (76)
Plastic surgery	451 970	160 (58)	432 110	116 (42)	0.96	485 160	50 (22)	1.12	401 720^c^	21 (9)	0.93	433 540	155 (69)
Urology	416 980	364 (73)	383 270	136 (27)	0.92	390 500	91 (22)	0.93	371 090	37 (9)	0.89	418 150	287 (69)
Ophthalmology	324 350	337 (47)	280 240	379 (53)	0.86	290 940	192 (32)	0.95	287 800	50 (8)	0.94	305 820	349 (59)
Otolaryngology	385 000	389 (60)	350 000	255 (40)	0.91	353 600	123 (24)	0.95	359 500	37 (7)	0.97	372 050	352 (69)

Abbreviation: URIM, underrepresented in medicine.

^a^ Overall includes all of the specialties reported in the Association of American Medical Colleges Faculty Salary Report, whereas total 19 clinical specialties includes only the 19 specialties focused on in the analysis.

^b^ Median values were used to calculate salary ratios. As a result, the median ratio of the total 19 clinical specialties might not fall within median ratios for individual specialties.

^c^ Fewer than 30 survey respondents in specialty.

**Table 2.** Median Salary and Salary Ratios for Assistant Professor Faculty Across Specialties by Gender-Race-Ethnicity

Specialty	Gender-race-ethnicity																
	Asian men			Asian women			URIM men			URIM women			White men		White women		
	Median, $	No. (%)	Ratio	Median, $	No. (%)	Ratio	Median, $	No. (%)	Ratio	Median, $	No. (%)	Ratio	Median, $	No. (%)	Median, $	No. (%)	Ratio
Overall ^a^	325 980	5054 (13)	0.98	265 720	5178 (13)	0.80	308 090	1961 (5)	0.92	259 570	2556 (7)	0.78	333 800	12 664 (33)	269 020	11 114 (29)	0.81
Total 19 clinical specialties^a^^,^^b^	336 670	4732 (13)	0.97	274 070	4881 (14)	0.79	305 730	1629 (5)	0.88	264 950	2227 (6)	0.77	345 960	11 760 (33)	281 430	10 420 (29)	0.82
**Nonsurgical specialties**																	
Anesthesiology	457 630	423 (16)	1.00	424 120	284 (10)	0.93	469 810	153 (6)	1.03	424 620	129 (5)	0.93	457 330	1118 (41)	433 010	617 (23)	0.95
Dermatology	411 810	30 (8)	1.03	348 410	52 (13)	0.87	342 480^c^	8 (2)	0.86	350 000^c^	7 (2)	0.88	398 740	106 (27)	364 890	196 (49)	0.92
Family medicine	254 580	115 (6)	1.00	236 810	196 (11)	0.93	244 100	119 (6)	0.96	231 340	210 (11)	0.91	255 460	613 (33)	232 650	601 (32)	0.91
Internal medicine	297 090	2027 (17)	1.02	257 180	1982 (17)	0.88	282 080	653 (5)	0.97	251 070	659 (6)	0.86	291 410	3625 (31)	250 840	2795 (24)	0.86
Clinical pathology	241 250	37 (13)	0.99	242 680	53 (18)	1.00	263 680^c^	8 (3)	1.08	243 590^c^	10 (3)	1.00	243 620	105 (36)	247 420	78 (27)	1.02
Pediatrics	242 460	423 (8)	1.02	216 620	900 (16)	0.91	231 920	155 (3)	0.97	210 870	492 (9)	0.88	238 720	1192 (21)	217 550	2405 (43)	0.91
Psychiatry	259 560	214 (12)	1.02	253 120	230 (13)	0.99	265 540	91 (5)	1.04	247 660	155 (8)	0.97	254 870	529 (29)	248 140	608 (33)	0.97
Radiology	439 550	447 (20)	0.95	422 750	242 (11)	0.92	448 520	92 (4)	0.97	434 810	74 (3)	0.94	461 120	926 (42)	428 690	412 (19)	0.93
Emergency medicine	331 400	152 (8)	1.02	306 160	124 (7)	0.94	318 340	90 (5)	0.98	308 020	103 (6)	0.94	326 000	851 (46)	313 920	542 (29)	0.96
Neurology	268 350	224 (16)	1.05	242 760	209 (15)	0.95	261 500	66 (5)	1.03	249 610	66 (5)	0.98	254 950	460 (33)	240 250	379 (27)	0.94
Physical medicine and rehabilitation	282 730	67 (14)	0.96	246 040	65 (13)	0.83	276 130	30 (6)	0.94	255 820	34 (7)	0.87	295 160	174 (35)	246 710	126 (25)	0.84
**Surgical specialties**																	
Obstetrics and gynecology	324 100	50 (3)	0.91	309 060	207 (12)	0.87	310 000	68 (4)	0.87	307 960	214 (13)	0.86	357 260	262 (15)	327 970	907 (53)	0.92
General surgery	409 000	77 (13)	0.95	351 680	62 (10)	0.82	372 940	30 (5)	0.87	420 600^c^	23 (4)	0.98	428 930	261 (44)	375 530	146 (24)	0.86
Neurosurgery	688 820	96 (24)	0.99	606 150	19 (5)	0.87	ND	ND	ND	ND	ND	ND	694 180	247 (62)	600 000	39 (10)	0.86
Orthopedic surgery	536 790	126 (14)	0.94	402 170^c^	25 (3)	0.71	500 870^c^	27 (3)	0.88	438 950^c^	14 (2)	0.77	568 310	583 (64)	478 170	136 (15)	0.84
Plastic surgery	468 880^c^	29 (14)	1.08	485 160^c^	20 (10)	1.12	ND	ND	ND	ND	ND	ND	433 540	83 (41)	425 000	71 (35)	0.98
Urology	401 080	60 (15)	0.94	382 850^c^	31 (8)	0.90	376 590^c^	12 (3)	0.88	351 550^c^	13 (3)	0.82	426 320	215 (53)	400 000	71 (18)	0.94
Ophthalmology	329 820	72 (13)	1.01	284 000	120 (21)	0.87	286 870^c^	11 (2)	0.88	247 470^c^	15 (3)	0.76	327 020	191 (34)	279 500	158 (28)	0.85
Otolaryngology	362 990	63 (13)	0.93	345 170	60 (12)	0.88	366 820^c^	16 (3)	0.94	348 170^c^	9 (2)	0.89	390 230	219 (44)	354 870	133 (27)	0.91

Abbreviations: ND, not determined; URIM, underrepresented in medicine.

^a^ Overall includes all of the specialties reported in the Association of American Medical Colleges Faculty Salary Report, whereas total 19 clinical specialties includes only the 19 specialties focused on in the analysis.

^b^ Median values were used to calculate salary ratios. As a result, the median ratio of the total 19 clinical specialties might not fall within median ratios for individual specialties.

^c^ Fewer than 30 survey respondents in specialty.

### Gender Disparities

**Figure 1.**
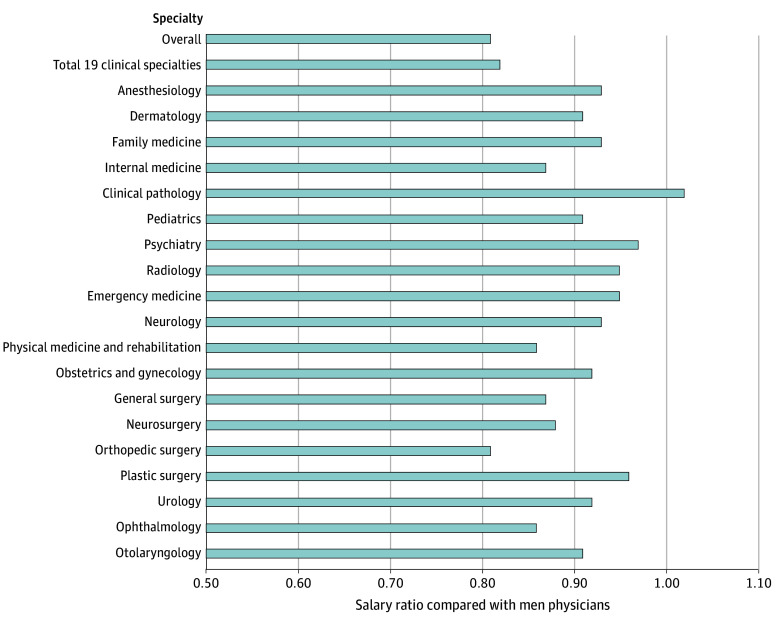
Gender Salary Differences Across Specialties Overall includes all specialties reported in the Association of American Medical Colleges Faculty Salary Report, whereas the total of 19 clinical specialties includes only the 19 specialties focused on in the analysis. A salary ratio less than 1.00 indicates that women earn less than men.

Across all specialties, women physicians (median annual salary, $266 450) were paid less than men (median annual salary, $330 000), with an overall salary ratio for women of $0.81:$1.00 (Table 1, Figure 1). The largest gender salary difference by ratio was in orthopedic surgery, with women receiving a median of $455 610 compared with men, who received $559 990 (absolute salary difference, $104 380; salary ratio $0.81:$1.00). This was followed by an absolute salary differences of $44 110 (ratio, $0.86:$1.00) for ophthalmology, and then $40 160 (ratio, $0.86:$1.00) for physical medicine and rehabilitation. Psychiatry (absolute salary difference, $6410; ratio, $0.97:$1.00) and plastic surgery ($19 860; ratio, $0.96:$1.00) had the smallest absolute gender salary differences. Clinical pathology was the only specialty in which women were paid more than men (absolute salary difference, $4070; ratio, $1.02:$1.00).

### Race and Ethnicity Disparities

**Figure 2.**
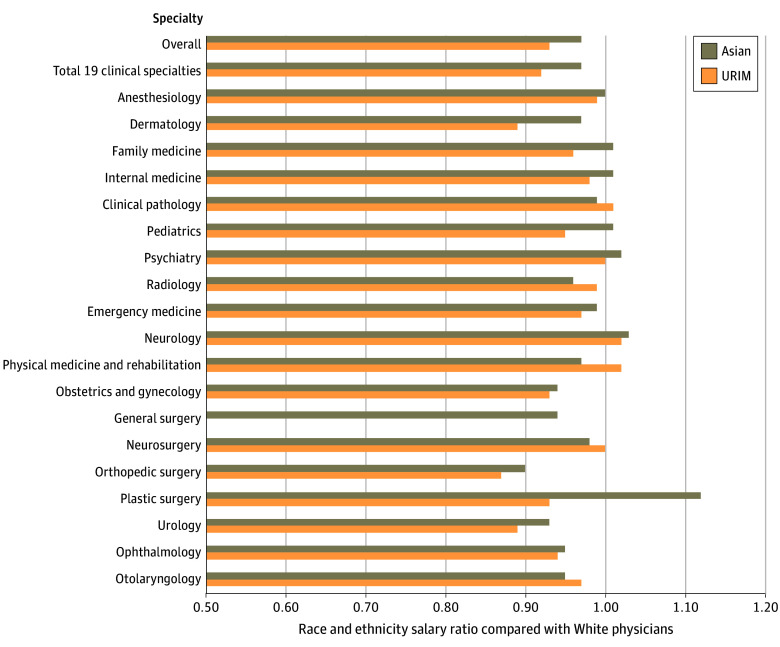
Race and Ethnicity Salary Differences Across Specialties Overall includes all specialties reported in the Association of American Medical Colleges Faculty Salary Report, whereas the total of 19 clinical specialties includes only the 19 specialties focused on in the analysis. A salary ratio less than 1.00 indicates that Asian and underrepresented in medicine (URIM) physicians earn less than White physicians.

Across all specialties, Asian (median annual salary, $291 360) and URIM (median annual salary, $278 010) physicians were paid less than White physicians (median annual salary, $300 000), with salary ratios of $0.97:$1.00 and $0.93:$1.00, respectively (Table 1, Figure 2). Asian physicians had the largest absolute salary differences in orthopedic surgery ($54 730; salary ratio, $0.90:$1.00), urology ($27 660; salary ratio, $0.93:$1.00), general surgery ($24 040; salary ratio, $0.94:$1.00), and obstetrics and gynecology ($19 040; salary ratio, $0.94:$1.00). Asian physicians were paid more than White physicians in 6 specialties; the only surgical specialty, plastic surgery, had the largest absolute salary difference ($51 620) and ratio ($1.12:$1.00).

URIM physicians had the largest absolute salary differences in orthopedic surgery ($73 410; salary ratio, $0.87:$1.00), urology ($47 070; salary ratio, $0.89:$1.00), and dermatology ($41 420; salary ratio, $0.89:$1.00). URIM physicians were paid as much or slightly more than White physicians in 5 specialties, with absolute salary differences of $5810 (salary ratio, $1.02:$1.00) for neurology, $515 (salary ratio, $1.02:$1.00) for physical medicine and rehabilitation, $2730 (salary ratio, $1.01:$1.00) for clinical pathology, $165 (salary ratio, $1.00:$1.00) for psychiatry, and $2360 (salary ratio, $1.00:$1.00) for neurosurgery. Plastic surgery was excluded because it had fewer than 30 observations.

### Gender-Race-Ethnicity Intersectionality Disparities

**Figure 3.**
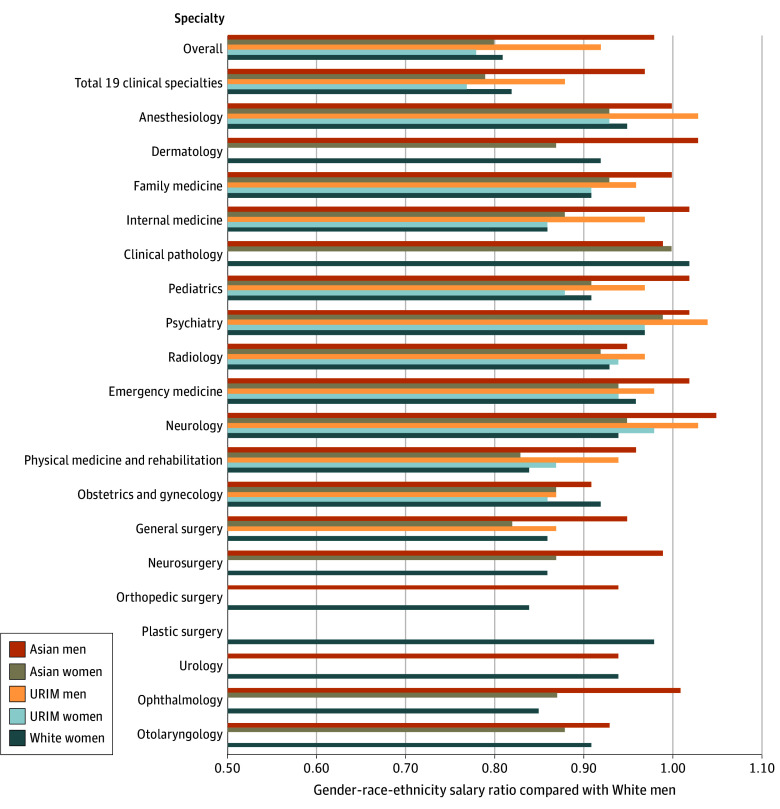
Gender-Race-Ethnicity Salary Differences Across Specialties Overall includes all specialties reported in the Association of American Medical Colleges Faculty Salary Report, whereas the total of 19 clinical specialties includes only the 19 specialties focused on in the analysis. A salary ratio less than 1.00 indicates that Asian and underrepresented in medicine (URIM) physicians and women physicians earn less than White physicians. If data were unavailable for a specific gender-race-ethnicity group within a specialty, no bar is shown for that group in the figure.

Asian men and women, URIM men and women, and White women all experienced salary differences compared with White men (Table 2, Figure 3). Across all specialties, URIM women experienced the largest salary differences, earning less than their counterparts (median annual salary, $259 570; absolute salary difference, $74 230; salary ratio, $0.78:$1.00), followed by Asian women (absolute salary difference, $68 080; salary ratio, $0.80:$1.00), White women (absolute salary difference, $64 780; salary ratio, $0.81:$1.00), URIM men (absolute salary difference, $25 710; salary ratio, $0.92:$1.00), then Asian men (absolute salary difference, $7820; salary ratio, $0.98:$1.00). There were no specialties in which the salaries of URIM women were equal to White men (median annual salary, $333 800). Asian men had salaries that were equal to or greater than White men in 8 specialties. Mean salary values are shown in the eTable 1 in Supplement 1 for comparison.

Asian men physicians had the largest salary difference compared with White men in obstetrics and gynecology (absolute salary difference, $33 160; salary ratio, $0.91:$1.00). They were paid equal to or greater than White men in 8 specialties, 7 of which were nonsurgical, with neurology being the specialty in which they earned the most compared with White men (absolute salary difference, $13 400; salary ratio, $1.05:$1.00). Asian women physicians experienced the largest salary difference in general surgery (absolute salary difference, $102 750; salary ratio, $0.82:$1.00) and matched White men only in clinical pathology (absolute salary difference, $941; salary ratio, $1.00:$1.00).

URIM men physicians experienced the largest salary differences compared with White men in obstetrics and gynecology and general surgery, in which they were paid a median of $310 000 (absolute salary difference, $47 260; salary ratio, $0.87:$1.00) and of $372 940 (absolute salary difference, $55 990; salary ratio, $0.87:$1.00), respectively, compared with White men, who were paid a median of $357 260 and of $428 930, respectively. URIM men were paid more than White men in 3 specialties, including anesthesiology (absolute salary difference, $12 480; salary ratio, $1.03:$1.00), psychiatry (absolute salary difference, $10 670; salary ratio, $1.04:$1.00), and neurology (absolute salary difference, $6553; salary ratio, $1.03:$1.00). For URIM women physicians, the largest salary differences were in internal medicine (absolute salary difference, $40 340; salary ratio, $0.86:$1.00) and obstetrics and gynecology (absolute salary difference, $49 305; salary ratio, $0.86:$1.00). URIM women physicians were paid comparable to White men in none of the 19 clinical specialties.

White women physicians had the largest salary difference in orthopedic surgery (absolute salary difference, $90 140; salary ratio, $0.84:$1.00). They were paid more than White men only in clinical pathology (absolute salary difference, $3800; salary ratio, $1.02:$1.00).

Gender-race-ethnicity data were limited for URIM and Asian physicians in surgical specialties. For URIM, 6 of 8 specialties for men and 7 for women had fewer than 30 respondents and were excluded. Neurosurgery and plastic surgery had 0 URIM respondents. For Asian men physicians, plastic surgery (29 of 203 [14%]) had fewer than 30 respondents, and for Asian women, 3 specialties had fewer than 30 respondents: orthopedic surgery (25 of 911 [3%]), plastic surgery (20 of 203 [10%]), and urology (12 of 402 [3%]) (Table 2, Figure 3).

### Specialty Representation and Salary Disparities

Across all specialties, we found no statistically significant association between salary differences and gender, race and ethnicity, and gender-race-ethnicity representation. Values for ρ ranged from −0.38 to 0.25 (*P* ranged from .28 to >.99) (eTable 2 in Supplement 2).

## Discussion

In this cross-sectional study of faculty compensation by gender, race and ethnicity, and gender-race-ethnicity, we found that salary disparities existed for women, Asian, and URIM assistant professor faculty across all specialties, and that the largest disparities existed at gender-race-ethnicity intersections. URIM women accounted for the highest salary disparities experienced across all specialties, earning $0.78 compared with every dollar earned by White men. Surgical specialties had the largest salary differences across gender and race and ethnicity groups; however, we were limited in our analysis for URIM men and women due to a lack of salary data. We also found that there was no association between specialty representation and salary disparities.

Our findings are consistent with pay disparities observed in the US workforce. Prior studies have shown that women physicians are paid less than men physicians.^6,7,8,9,10,11,12^ Our findings extend prior work by highlighting inequities at gender-race-ethnicity intersections. For example, Asian women and URIM women accounted for the highest salary disparities experienced across all specialties, earning $0.80 and $0.78, respectively, compared with every dollar earned by White men. This difference was larger than those observed in nonintersectional analyses, suggesting that race augments gender pay disparities in ways not previously described, and that nonintersectional analyses may overlook important workforce pay disparities.

Moreover, our analysis did not find an association between demographic composition of a specialty and the magnitude of salary differences among assistant professors. This finding underscores that simply increasing diversity does not, in itself, guarantee pay equity. Achieving pay equity requires a more comprehensive approach, including promoting salary transparency, prioritizing pay equity in initial salary offers for new hires, conducting salary reviews for current employees through an equity lens, and implementing training programs to address implicit bias.^21^ Several factors may contribute to the lack of an association. Our study was potentially underpowered to detect subtle associations due to sample size limitations, particularly within individual specialties. Furthermore, data constraints, especially the limited data for Asian and URIM physicians in certain surgical specialties, may have influenced our findings. While true equity may exist in some settings, the persistent descriptive salary disparities observed in our study suggest that this is unlikely to be the sole explanation. Further research with larger sample sizes and more comprehensive data are needed to fully understand the association between specialty diversity and salary equity.

This study highlights the need for improved salary equity and representation data for Asian and URIM men and women physicians, particularly in surgical specialties. By addressing these data gaps and implementing targeted interventions, academic institutions can work toward a more equitable and inclusive environment for all faculty members.

### Limitations

This study has limitations that should be considered when interpreting the findings. First, the AAMC reports aggregated data; thus, we were unable to account for possible disparities experienced within racial and ethnic subgroups. For instance, in Asian ethnic subgroups, significant salary differences exist in the labor workforce. Indian Americans, for example, have a median household income that is 3 times higher than that of Burmese Americans.^22,23,24^ Other ethnic differences likely exist among Black and Latinx subgroups.^25,26^ As a result, in our study, aggregation of data limited our ability to observe any potential disparities that existed within racial and ethnic groups. In addition, aggregated data do not allow for the adjustment of other factors that may affect salary, such as age, years in practice, geographic location, fellowship training, or clinical or scholarly productivity.

Second, the absence of salary data for URIM men and women, especially across surgical specialties, restricted our ability to draw firm conclusions about salary equity for these groups in those specialties. Notably, this absence reflects the underrepresentation of Asian and URIM physicians, especially women, at the assistant professor level in those specialties, highlighting the need for targeted initiatives to improve representation.

There are several barriers to obtaining and reporting more complete data, most notably the underrepresentation of URIM and Asian physicians, particularly women, in academic positions, especially within surgical specialties. To address these limitations in future research, we recommend several strategies. The first is to implement targeted initiatives to increase the recruitment and retention of Asian and URIM physicians in academic medicine. The second is to explore alternative reporting methods that could provide more granular data without compromising individual privacy. Third, we recommend the continuation of qualitative research, such as interviews and focus groups, to gain deeper insights into the experiences and perceptions of Asian and URIM physicians regarding salary equity.

Additionally, our cross-sectional study design limited our ability to examine salary trends over time or to consider any potential salary disparities at the associate or full professor levels. Furthermore, the AAMC’s definition of compensation may not fully capture all sources of income, potentially underreporting compensation. However, we anticipate that this would affect all groups similarly.

## Conclusions

In this cross-sectional study of assistant professor faculty, Asian and URIM men and women were paid less than White men across clinical specialties in the academic year 2022-2023. Limited race and ethnicity data, particularly for URIM faculty in surgical specialties, impacted the generalizability of our findings. Salary disparities were not mitigated by specialty diversity. Further research is needed to understand the causes of these salary differences and to develop interventions for equitable compensation in academic medicine, with a focus on intersectional identities.

## Notes

Supplement 1. **eTable 1.** Mean Salary Values and Ratios Calculated Based on Mean Values

Supplement 2. **eTable 2.** Gender, Race and Ethnicity, and Gender-Race-Ethnicity Composition Across Specialties, and Their Associations With Salary Differences

Supplement 3.
Data Sharing Statement
