# Supplementary material for: Salary Differences by Gender, Race, and Ethnicity Among Assistant Professors at US Medical Schools
Source: JAMA Netw Open. 2025 May 14;8(5):e259583. doi: 10.1001/jamanetworkopen.2025.9583 (PMC12079292; doi:10.1001/jamanetworkopen.2025.9583)
Supplement: Supplement 2. — eTable 2. Gender, Race and Ethnicity, and Gender-Race-Ethnicity Composition Across Specialties, and Their Associations With Salary Differences [file jamanetwopen-e259583-s002.pdf]

Supplemental Online Content

Owda D, Mensah MO, Yang D, Canavan ME, Gross CP, Chaudhry SI. Salary Differences by gender, race, and ethnicity among assistant professors at US medical schools. *JAMA Netw Open*. 2025;8(5):e259583. doi:10.1001/jamanetworkopen.2025.9583

eTable 2. Gender, Race and Ethnicity, and Gender-Race-Ethnicity Composition Across Specialties, and Their Associations With Salary Differences

This supplemental material has been provided by the authors to give readers additional information about their work.

© 2025 Owda D et al. *JAMA Network Open*.

eTable 2. Gender, Race and Ethnicity, and Gender-Race-Ethnicity Composition Across Specialties, and Their Associations With Salary Differences

Specialty	Respondents, No. (%)										
	Gender		Race and ethnicity			Gender-race-ethnicity					
	**Men**	**Women**	**Asian**	**URIM**	**White**	**Asian men**	**Asian women**	**URIM men**	**URIM women**	**White men**	**White women**
Overall	43 535 (52)	41 031 (48)	48 932 (24)	10 082 (12)	48 932 (58)	10 136 (12)	9756 (12)	4653 (6)	5429 (6)	25 919 (31)	23 013 (27)
**Nonsurgical specialties**											
Anesthesiology	3131 (62)	1910 (38)	1126 (22)	595 (12)	3032 (60)	665 (13)	461 (9)	309 (6)	286 (6)	1966 (39)	1066 (21)
Dermatology	281 (38)	448 (62)	139 (19)	75 (10)	476 (65)	54 (7)	85 (12)	27 (4)	48 (7)	181 (25)	295 (40)
Family medicine	1772 (45)	2185 (55)	619 (16)	615 (16)	2438 (62)	248 (6)	371 (9)	236 (6)	379 (10)	1172 (30)	1266 (32)
Internal medicine	12 492 (54)	10 488 (46)	7228 (32)	2638 (12)	11 626 (51)	3742 (16)	3486 (15)	1366 (6)	1272 (6)	6542 (28)	5084 (22)
Clinical pathology	938 (49)	969 (51)	549 (29)	184 (10)	1037 (54)	252 (13)	297 (16)	82 (4)	102 (5)	531 (28)	506 (26)
Pediatrics	3931 (32)	8236 (68)	2533 (201)	1653 (14)	7268 (60)	860 (7)	1673 (14)	458 (4)	1195 (10)	2401 (20)	4867 (40)
Psychiatry	2542 (40)	3767 (60)	1002 (16)	777 (12)	3896 (62)	457 (7)	545 (9)	285 (4)	492 (8)	1570 (25)	2326 (37)
Radiology	3394 (68)	1607 (32)	1412 (28)	410 (8)	2832 (57)	930 (19)	482 (10)	254 (5)	156 (3)	1972 (39)	860 (17)
Emergency medicine	2255 (58)	1604 (42)	522 (14)	477 (12)	2673 (70)	280 (7)	242 (6)	243 (6)	234 (6)	1626 (42)	1047 (27)
Neurology	1533 (50)	1537 (50)	863 (28)	303 (10)	1698 (55)	424 (14)	439 (14)	155 (5)	148 (5)	855 (28)	843 (28)
Physical medicine and rehabilitation	553 (49)	583 (51)	232 (20)	114 (10)	656 (58)	110 (10)	122 (11)	55 (5)	59 (5)	336 (30)	320 (28)
**Surgical specialties**											
Obstetrics and gynecology	878 (26)	2571 (74)	463 (13)	605 (18)	2210 (64)	112 (3)	351 (10)	154 (4)	451 (13)	576 (16.7)	1634 (47)
General surgery	5461 (69)	2487 (31)	1553 (20)	894 (11)	4997 (63)	1022 (13)	531 (7)	598 (8)	296 (4)	3516 (44.2)	1481 (19)
Neurosurgery	ND	ND	ND	ND	ND	ND	ND	ND	ND	ND	ND
Orthopedic surgery	1532 (77)	466 (23)	290 (14)	148 (7)	1456 (73)	218 (11)	72 (4)	114 (6)	34 (2)	1129 (56.5)	327 (16)
Plastic surgery	ND	ND	ND	ND	ND	ND	ND	ND	ND	ND	ND
Urology	ND	ND	ND	ND	ND	ND	ND	ND	ND	ND	ND
Ophthalmology	626 (50)	629 (50)	410 (33)	100 (8)	659 (52)	182 (14)	228 (18)	38 (3)	62 (5)	370 (29.5)	289 (23)
Otolaryngology	583 (60)	389 (40)	191 (20)	78 (8)	638 (66)	115 (12)	76 (8)	39 (4)	39 (4)	391 (40.2)	247 (25)
**Associations with salary differences**											
Correlation coefficient[a]	NA	0.18	0.20	−0.10	NA	0	−0.01	−0.07	−0.38	NA	0.25
P value[b]	NA	.49	.40	.80	NA	>.99	.96	.84	.28	NA	.35
No. of specialties in analysis	NA	16	16	15	NA	16	15	11	10	NA	16

Abbreviations: NA, not applicable; ND, not determined; URIM, underrepresented in medicine.

[a]A Spearman correlation coefficient was calculated to measure the association between a group's representation and their salary differences, denoted by the salary ratio. A correlation coefficient with an absolute value between 0 and 0.3 indicates a weak correlation; between 0.3 and 0.7, a moderate correlation; and >0.7, a strong correlation.

[b]$P \leq .05$ was considered statistically significant.

© 2025 Owda D et al. *JAMA Network Open*.
