# Supplementary material for: Salary Differences by Gender, Race, and Ethnicity Among Assistant Professors at US Medical Schools
Source: JAMA Netw Open. 2025 May 14;8(5):e259583. doi: 10.1001/jamanetworkopen.2025.9583 (PMC12079292; doi:10.1001/jamanetworkopen.2025.9583)
Supplement: Supplement 3. — Data Sharing Statement [file jamanetwopen-e259583-s003.pdf]

Data Sharing Statement

Owda. Salary Differences by Gender, Race, and Ethnicity Among Assistant Professors at US Medical Schools. *JAMA Netw Open*. Published May 14, 2025.
doi:10.1001/jamanetworkopen.2025.9583

Data
Data available: No

Additional Information
Explanation for why data not available: It is publically available data through the AAMC.
